# 1,3-Dimethyl-5-(3-methyl­phen­oxy)-1*H*-pyrazole-4-carbaldehyde

**DOI:** 10.1107/S1600536811050926

**Published:** 2011-11-30

**Authors:** Hong Dai, Hai-Jun Zhang, Lei Shi, Hai-Qin Sun, Yu-Jun Shi

**Affiliations:** a College of Chemistry and Chemical Engineering, Nantong University, Nantong 226019, People’s Republic of China

## Abstract

There are two independent mol­ecules in the asymmetric unit of the title compound, C_13_H_14_N_2_O_2_, in which the dihedral angles between the substituted phenyl ring and the pyrazole ring are 86.5 (2) and 82.3 (3)°. The crystal packing features weak inter­molecular C—H⋯O inter­actions.

## Related literature

For the biological activity of pyrazole derivatives, see: Drabek (1992 ▶); Haga *et al.* (1990 ▶); Motoba *et al.* (1992 ▶); Watanabe *et al.* (2001 ▶).
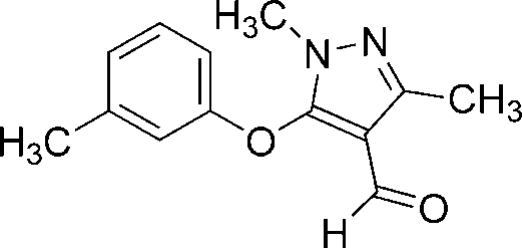


## Experimental

### 

#### Crystal data

C_13_H_14_N_2_O_2_
*M* *_r_* = 230.26
Triclinic, 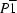

*a* = 7.9444 (16) Å
*b* = 10.643 (3) Å
*c* = 15.053 (3) Å
α = 107.732 (3)°
β = 102.473 (5)°
γ = 93.225 (7)°
*V* = 1173.4 (5) Å^3^
*Z* = 4
Mo *K*α radiation
μ = 0.09 mm^−1^
*T* = 113 K
0.20 × 0.16 × 0.12 mm

#### Data collection

Rigaku Saturn724 CCD diffractometer
Absorption correction: multi-scan (*CrystalClear*; Rigaku, 2008 ▶) *T*_min_ = 0.982, *T*_max_ = 0.989
12386 measured reflections
5529 independent reflections
2226 reflections with *I* > 2σ(*I*)
*R*_int_ = 0.064

#### Refinement

*R*[*F* ^2^ > 2σ(*F* ^2^)] = 0.057
*wR*(*F* ^2^) = 0.100
*S* = 1.02
5529 reflections
313 parameters
H-atom parameters constrained
Δρ_max_ = 0.25 e Å^−3^
Δρ_min_ = −0.40 e Å^−3^

### 

Data collection: *CrystalClear* (Rigaku, 2008 ▶); cell refinement: *CrystalClear*; data reduction: *CrystalClear*; program(s) used to solve structure: *SHELXS97* (Sheldrick, 2008 ▶); program(s) used to refine structure: *SHELXL97* (Sheldrick, 2008 ▶); molecular graphics: *SHELXTL* (Sheldrick, 2008 ▶); software used to prepare material for publication: *SHELXTL*.

## Supplementary Material

Crystal structure: contains datablock(s) global, I. DOI: 10.1107/S1600536811050926/ds2155sup1.cif

Structure factors: contains datablock(s) I. DOI: 10.1107/S1600536811050926/ds2155Isup2.hkl

Supplementary material file. DOI: 10.1107/S1600536811050926/ds2155Isup3.cml

Additional supplementary materials:  crystallographic information; 3D view; checkCIF report

## Figures and Tables

**Table 1:** Hydrogen-bond geometry (Å, °)

*D*—H⋯*A*	*D*—H	H⋯*A*	*D*⋯*A*	*D*—H⋯*A*
C12—H12*A*⋯O4^i^	0.98	2.57	3.488 (3)	157
C15—H15⋯O2^ii^	0.95	2.58	3.315 (3)	134

Symmetry codes: (i) 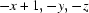; (ii) 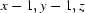.

**Fig. 1.**
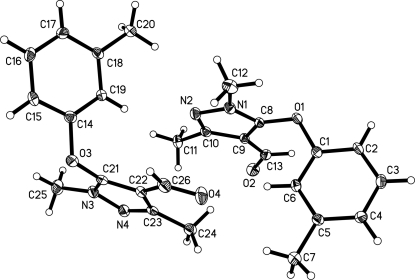
View of the title compound (I), with displacement ellipsoids drawn at the 30% probability level.

## supplementary crystallographic information

### Comment

It is well known that compounds containing pyrazole ring have good bioactivities such as fungicidal, insecticidal, and herbicidal activities (Haga *et al.*, 1990; Motoba *et al.*, 1992; Watanabe *et al.*, 2001). They are widely applied in the field of plant protection (Drabek, 1992). In order to discover more biologically active pyrazole compounds, the title compound, (I), was synthesized and its crystal structure was determined (Fig.1). The dihedral angles between substituted phenyl ring and pyrazole ring in the two independent molecules are 86.5 (2) ° and 82.3 (3) °, respectively. The crystal packing displays weak intermolecular C—H···O interactions (Table 1).

### Experimental

To a stirred solution of 1-methyl-3-methyl-5-chloro-1*H*-pyrazole- 4-carbaldehyde(30 mmol) and 3-methylphenol(48 mmol) in DMF(30 ml) was added potassium hydroxide(60 mmol) at room temperature. The resulting mixture was heated to 388 k for 6 h. Then the reaction solution was poured into cold water(100 ml) and extracted with ethyl acetate (3 *x* 60 ml). The organic layer was dried over anhydrous magnesium sulfate. After removal of the solvent, the residue was recrystallized from ethyl acetate/petroleum ether to give colourless crystals.

### Refinement

All H atoms were placed in calculated positions, with C–H = 0.95, and 0.98 ° A, and included in the final cycles of refinement using a riding model, with *U*_iso_(H) = 1.2*U*_eq_(C).

### Crystal data

**Table:**

C_13_H_14_N_2_O_2_	*Z* = 4
*M**_r_* = 230.27	*F*(000) = 488
Triclinic, *P*1	*D*_x_ = 1.303 Mg m^−^^3^
Hall symbol: -P 1	Mo *K*α radiation, λ = 0.71073 Å
*a* = 7.9444 (16) Å	Cell parameters from 4226 reflections
*b* = 10.643 (3) Å	θ = 2.0–28.1°
*c* = 15.053 (3) Å	µ = 0.09 mm^−^^1^
α = 107.732 (3)°	*T* = 113 K
β = 102.473 (5)°	Prism, colourless
γ = 93.225 (7)°	0.20 × 0.16 × 0.12 mm
*V* = 1173.4 (5) Å^3^	

### Data collection

**Table:**

Rigaku Saturn724 CCD diffractometer	5529 independent reflections
Radiation source: rotating anode	2226 reflections with *I* > 2σ(*I*)
multilayer	*R*_int_ = 0.064
Detector resolution: 14.22 pixels mm^-1^	θ_max_ = 28.0°, θ_min_ = 2.0°
ω and φ scans	*h* = −10→10
Absorption correction: multi-scan (*CrystalClear*; Rigaku, 2008)	*k* = −14→14
*T*_min_ = 0.982, *T*_max_ = 0.989	*l* = −19→19
12386 measured reflections	

### Refinement

**Table:**

Refinement on *F*^2^	Primary atom site location: structure-invariant direct methods
Least-squares matrix: full	Secondary atom site location: difference Fourier map
*R*[*F*^2^ > 2σ(*F*^2^)] = 0.057	Hydrogen site location: inferred from neighbouring sites
*wR*(*F*^2^) = 0.100	H-atom parameters constrained
*S* = 1.02	*w* = 1/[σ^2^(*F*_o_^2^) + (0.010*P*)^2^] where *P* = (*F*_o_^2^ + 2*F*_c_^2^)/3
5529 reflections	(Δ/σ)_max_ = 0.004
313 parameters	Δρ_max_ = 0.25 e Å^−^^3^
0 restraints	Δρ_min_ = −0.40 e Å^−^^3^

### Special details

**Table:**

Geometry. All e.s.d.'s (except the e.s.d. in the dihedral angle between two l.s. planes) are estimated using the full covariance matrix. The cell e.s.d.'s are taken into account individually in the estimation of e.s.d.'s in distances, angles and torsion angles; correlations between e.s.d.'s in cell parameters are only used when they are defined by crystal symmetry. An approximate (isotropic) treatment of cell e.s.d.'s is used for estimating e.s.d.'s involving l.s. planes.
Refinement. Refinement of *F*^2^ against ALL reflections. The weighted *R*-factor *wR* and goodness of fit *S* are based on *F*^2^, conventional *R*-factors *R* are based on *F*, with *F* set to zero for negative *F*^2^. The threshold expression of *F*^2^ > σ(*F*^2^) is used only for calculating *R*-factors(gt) *etc*. and is not relevant to the choice of reflections for refinement. *R*-factors based on *F*^2^ are statistically about twice as large as those based on *F*, and *R*- factors based on ALL data will be even larger.

### Fractional atomic coordinates and isotropic or equivalent isotropic displacement parameters (Å^2^)

**Table:**

	*x*	*y*	*z*	*U*_iso_*/*U*_eq_	
O1	0.8036 (2)	0.24450 (16)	0.10860 (11)	0.0294 (5)	
O2	0.7737 (2)	0.60698 (17)	0.33053 (12)	0.0382 (5)	
O3	0.0347 (2)	0.00932 (17)	0.28476 (11)	0.0328 (5)	
O4	0.2005 (2)	0.11940 (17)	0.05903 (12)	0.0425 (5)	
N1	0.6563 (3)	0.1553 (2)	0.19874 (14)	0.0278 (6)	
N2	0.5999 (3)	0.1925 (2)	0.28185 (14)	0.0294 (6)	
N3	0.0942 (3)	0.2408 (2)	0.35725 (15)	0.0303 (6)	
N4	0.1457 (3)	0.3542 (2)	0.33896 (14)	0.0297 (6)	
C1	0.7095 (3)	0.2757 (2)	0.02854 (17)	0.0241 (6)	
C2	0.8041 (3)	0.2834 (2)	−0.03652 (16)	0.0274 (7)	
H2	0.9235	0.2712	−0.0258	0.033*	
C3	0.7187 (3)	0.3095 (2)	−0.11844 (17)	0.0321 (7)	
H3	0.7797	0.3156	−0.1651	0.038*	
C4	0.5444 (3)	0.3268 (2)	−0.13217 (17)	0.0301 (7)	
H4	0.4875	0.3453	−0.1883	0.036*	
C5	0.4513 (3)	0.3177 (2)	−0.06618 (17)	0.0255 (6)	
C6	0.5371 (3)	0.2912 (2)	0.01631 (16)	0.0256 (6)	
H6	0.4767	0.2840	0.0630	0.031*	
C7	0.2610 (3)	0.3341 (2)	−0.08065 (16)	0.0341 (7)	
H7A	0.2039	0.2766	−0.0529	0.051*	
H7B	0.2067	0.3093	−0.1496	0.051*	
H7C	0.2488	0.4271	−0.0488	0.051*	
C8	0.7308 (3)	0.2600 (3)	0.18420 (17)	0.0256 (7)	
C9	0.7234 (3)	0.3731 (2)	0.25874 (17)	0.0223 (6)	
C10	0.6415 (3)	0.3234 (3)	0.31759 (17)	0.0255 (6)	
C11	0.5998 (3)	0.4000 (2)	0.40987 (15)	0.0320 (7)	
H11A	0.5401	0.3387	0.4337	0.048*	
H11B	0.5243	0.4661	0.3986	0.048*	
H11C	0.7077	0.4452	0.4577	0.048*	
C12	0.6303 (3)	0.0156 (2)	0.13985 (17)	0.0377 (8)	
H12A	0.6849	0.0053	0.0860	0.056*	
H12B	0.5054	−0.0155	0.1149	0.056*	
H12C	0.6830	−0.0372	0.1791	0.056*	
C13	0.7929 (3)	0.5060 (3)	0.26952 (18)	0.0313 (7)	
H13	0.8587	0.5164	0.2259	0.038*	
C14	0.1617 (3)	−0.0619 (3)	0.32283 (16)	0.0274 (7)	
C15	0.0930 (3)	−0.1703 (2)	0.34139 (16)	0.0294 (7)	
H15	−0.0289	−0.1912	0.3313	0.035*	
C16	0.2095 (3)	−0.2481 (2)	0.37553 (16)	0.0298 (7)	
H16	0.1670	−0.3246	0.3881	0.036*	
C17	0.3865 (3)	−0.2146 (2)	0.39111 (16)	0.0296 (7)	
H17	0.4643	−0.2684	0.4145	0.035*	
C18	0.4526 (3)	−0.1037 (2)	0.37326 (16)	0.0252 (6)	
C19	0.3373 (3)	−0.0259 (2)	0.33883 (15)	0.0249 (6)	
H19	0.3792	0.0510	0.3266	0.030*	
C20	0.6461 (3)	−0.0664 (2)	0.39066 (16)	0.0320 (7)	
H20A	0.6958	−0.0195	0.4593	0.048*	
H20B	0.6672	−0.0084	0.3535	0.048*	
H20C	0.7009	−0.1472	0.3704	0.048*	
C21	0.0920 (3)	0.1315 (3)	0.28273 (19)	0.0287 (7)	
C22	0.1413 (3)	0.1701 (3)	0.21237 (18)	0.0250 (6)	
C23	0.1758 (3)	0.3107 (3)	0.25212 (18)	0.0268 (7)	
C24	0.2314 (3)	0.4080 (2)	0.20729 (17)	0.0327 (7)	
H24A	0.2286	0.4987	0.2485	0.049*	
H24B	0.1522	0.3915	0.1440	0.049*	
H24C	0.3499	0.3978	0.1999	0.049*	
C25	0.0481 (3)	0.2495 (3)	0.44694 (16)	0.0417 (8)	
H25A	−0.0006	0.1613	0.4440	0.063*	
H25B	−0.0385	0.3111	0.4571	0.063*	
H25C	0.1521	0.2821	0.5003	0.063*	
C26	0.1513 (3)	0.0825 (3)	0.11980 (19)	0.0350 (7)	
H26	0.1175	−0.0100	0.1050	0.042*	

### Atomic displacement parameters (Å^2^)

**Table:**

	*U*^11^	*U*^22^	*U*^33^	*U*^12^	*U*^13^	*U*^23^
O1	0.0228 (11)	0.0443 (12)	0.0271 (10)	0.0118 (9)	0.0112 (8)	0.0153 (9)
O2	0.0450 (13)	0.0302 (12)	0.0371 (11)	0.0036 (10)	0.0104 (10)	0.0076 (10)
O3	0.0224 (11)	0.0375 (12)	0.0437 (12)	0.0048 (9)	0.0077 (9)	0.0208 (10)
O4	0.0511 (14)	0.0431 (13)	0.0393 (12)	0.0058 (10)	0.0263 (10)	0.0116 (10)
N1	0.0281 (14)	0.0296 (14)	0.0244 (12)	0.0053 (11)	0.0086 (11)	0.0055 (11)
N2	0.0295 (14)	0.0351 (14)	0.0256 (13)	0.0052 (11)	0.0107 (11)	0.0100 (11)
N3	0.0255 (14)	0.0421 (15)	0.0279 (13)	0.0077 (12)	0.0114 (11)	0.0140 (12)
N4	0.0258 (14)	0.0330 (14)	0.0307 (13)	0.0028 (11)	0.0083 (11)	0.0100 (12)
C1	0.0238 (17)	0.0238 (15)	0.0240 (14)	0.0034 (12)	0.0067 (12)	0.0063 (12)
C2	0.0215 (16)	0.0336 (17)	0.0306 (15)	0.0078 (13)	0.0135 (12)	0.0099 (13)
C3	0.0399 (19)	0.0322 (17)	0.0311 (15)	0.0071 (14)	0.0191 (13)	0.0126 (14)
C4	0.0325 (18)	0.0335 (17)	0.0271 (15)	0.0107 (14)	0.0082 (13)	0.0122 (13)
C5	0.0249 (17)	0.0253 (15)	0.0266 (15)	0.0062 (12)	0.0077 (12)	0.0074 (13)
C6	0.0268 (17)	0.0283 (16)	0.0229 (14)	0.0044 (13)	0.0108 (12)	0.0066 (12)
C7	0.0307 (18)	0.0389 (18)	0.0353 (16)	0.0066 (14)	0.0093 (13)	0.0143 (14)
C8	0.0182 (16)	0.0387 (18)	0.0262 (15)	0.0080 (13)	0.0074 (13)	0.0172 (14)
C9	0.0176 (15)	0.0266 (15)	0.0226 (14)	0.0014 (12)	0.0045 (12)	0.0086 (12)
C10	0.0200 (16)	0.0331 (16)	0.0229 (14)	0.0057 (13)	0.0042 (12)	0.0089 (13)
C11	0.0333 (17)	0.0374 (18)	0.0274 (15)	0.0030 (14)	0.0147 (13)	0.0086 (14)
C12	0.0386 (19)	0.0305 (17)	0.0364 (16)	0.0054 (14)	0.0074 (14)	0.0014 (14)
C13	0.0240 (17)	0.0394 (18)	0.0343 (17)	0.0042 (14)	0.0048 (13)	0.0190 (15)
C14	0.0274 (18)	0.0315 (17)	0.0240 (15)	0.0078 (13)	0.0080 (13)	0.0079 (13)
C15	0.0255 (17)	0.0360 (17)	0.0284 (15)	0.0030 (14)	0.0138 (13)	0.0083 (13)
C16	0.0387 (19)	0.0278 (16)	0.0275 (15)	0.0036 (14)	0.0160 (13)	0.0103 (13)
C17	0.0342 (18)	0.0299 (16)	0.0302 (15)	0.0112 (14)	0.0129 (14)	0.0131 (13)
C18	0.0238 (16)	0.0290 (16)	0.0224 (14)	0.0073 (13)	0.0088 (12)	0.0052 (13)
C19	0.0223 (16)	0.0290 (16)	0.0272 (14)	0.0047 (12)	0.0087 (12)	0.0126 (13)
C20	0.0308 (18)	0.0351 (17)	0.0356 (16)	0.0095 (13)	0.0132 (13)	0.0150 (14)
C21	0.0162 (16)	0.0374 (18)	0.0376 (17)	0.0087 (13)	0.0064 (13)	0.0188 (15)
C22	0.0214 (16)	0.0289 (16)	0.0281 (15)	0.0073 (13)	0.0097 (12)	0.0107 (13)
C23	0.0166 (16)	0.0350 (17)	0.0305 (15)	0.0082 (13)	0.0049 (12)	0.0130 (14)
C24	0.0309 (17)	0.0314 (17)	0.0380 (16)	0.0083 (14)	0.0116 (13)	0.0116 (14)
C25	0.046 (2)	0.057 (2)	0.0274 (16)	0.0085 (16)	0.0162 (14)	0.0173 (15)
C26	0.0320 (18)	0.0327 (18)	0.0411 (18)	0.0060 (14)	0.0140 (15)	0.0092 (15)

### Geometric parameters (Å, °)

**Table:**

O1—C8	1.356 (3)	C11—H11A	0.9800
O1—C1	1.415 (2)	C11—H11B	0.9800
O2—C13	1.224 (3)	C11—H11C	0.9800
O3—C21	1.364 (3)	C12—H12A	0.9800
O3—C14	1.422 (3)	C12—H12B	0.9800
O4—C26	1.224 (3)	C12—H12C	0.9800
N1—C8	1.330 (3)	C13—H13	0.9500
N1—N2	1.372 (3)	C14—C19	1.374 (3)
N1—C12	1.458 (3)	C14—C15	1.378 (3)
N2—C10	1.325 (3)	C15—C16	1.395 (3)
N3—C21	1.344 (3)	C15—H15	0.9500
N3—N4	1.377 (3)	C16—C17	1.382 (3)
N3—C25	1.451 (3)	C16—H16	0.9500
N4—C23	1.326 (3)	C17—C18	1.387 (3)
C1—C6	1.369 (3)	C17—H17	0.9500
C1—C2	1.375 (3)	C18—C19	1.392 (3)
C2—C3	1.388 (3)	C18—C20	1.511 (3)
C2—H2	0.9500	C19—H19	0.9500
C3—C4	1.386 (3)	C20—H20A	0.9800
C3—H3	0.9500	C20—H20B	0.9800
C4—C5	1.382 (3)	C20—H20C	0.9800
C4—H4	0.9500	C21—C22	1.367 (3)
C5—C6	1.398 (3)	C22—C23	1.418 (3)
C5—C7	1.508 (3)	C22—C26	1.443 (3)
C6—H6	0.9500	C23—C24	1.492 (3)
C7—H7A	0.9800	C24—H24A	0.9800
C7—H7B	0.9800	C24—H24B	0.9800
C7—H7C	0.9800	C24—H24C	0.9800
C8—C9	1.388 (3)	C25—H25A	0.9800
C9—C10	1.408 (3)	C25—H25B	0.9800
C9—C13	1.437 (3)	C25—H25C	0.9800
C10—C11	1.498 (3)	C26—H26	0.9500
			
C8—O1—C1	117.58 (19)	H12A—C12—H12C	109.5
C21—O3—C14	117.0 (2)	H12B—C12—H12C	109.5
C8—N1—N2	111.3 (2)	O2—C13—C9	125.1 (3)
C8—N1—C12	128.9 (2)	O2—C13—H13	117.5
N2—N1—C12	119.8 (2)	C9—C13—H13	117.5
C10—N2—N1	105.0 (2)	C19—C14—C15	123.2 (2)
C21—N3—N4	110.9 (2)	C19—C14—O3	122.7 (2)
C21—N3—C25	128.5 (2)	C15—C14—O3	114.0 (2)
N4—N3—C25	120.6 (2)	C14—C15—C16	117.4 (2)
C23—N4—N3	104.8 (2)	C14—C15—H15	121.3
C6—C1—C2	123.6 (2)	C16—C15—H15	121.3
C6—C1—O1	122.0 (2)	C17—C16—C15	120.3 (2)
C2—C1—O1	114.3 (2)	C17—C16—H16	119.9
C1—C2—C3	117.5 (2)	C15—C16—H16	119.9
C1—C2—H2	121.2	C16—C17—C18	121.2 (2)
C3—C2—H2	121.2	C16—C17—H17	119.4
C4—C3—C2	120.0 (2)	C18—C17—H17	119.4
C4—C3—H3	120.0	C17—C18—C19	118.8 (2)
C2—C3—H3	120.0	C17—C18—C20	121.3 (2)
C5—C4—C3	121.6 (2)	C19—C18—C20	119.8 (2)
C5—C4—H4	119.2	C14—C19—C18	119.0 (2)
C3—C4—H4	119.2	C14—C19—H19	120.5
C4—C5—C6	118.5 (2)	C18—C19—H19	120.5
C4—C5—C7	122.3 (2)	C18—C20—H20A	109.5
C6—C5—C7	119.2 (2)	C18—C20—H20B	109.5
C1—C6—C5	118.7 (2)	H20A—C20—H20B	109.5
C1—C6—H6	120.6	C18—C20—H20C	109.5
C5—C6—H6	120.6	H20A—C20—H20C	109.5
C5—C7—H7A	109.5	H20B—C20—H20C	109.5
C5—C7—H7B	109.5	N3—C21—O3	119.8 (2)
H7A—C7—H7B	109.5	N3—C21—C22	108.6 (2)
C5—C7—H7C	109.5	O3—C21—C22	131.5 (3)
H7A—C7—H7C	109.5	C21—C22—C23	104.0 (2)
H7B—C7—H7C	109.5	C21—C22—C26	125.8 (3)
N1—C8—O1	120.9 (2)	C23—C22—C26	130.2 (3)
N1—C8—C9	108.3 (2)	N4—C23—C22	111.7 (2)
O1—C8—C9	130.8 (3)	N4—C23—C24	119.8 (2)
C8—C9—C10	103.7 (2)	C22—C23—C24	128.5 (2)
C8—C9—C13	125.1 (3)	C23—C24—H24A	109.5
C10—C9—C13	131.2 (2)	C23—C24—H24B	109.5
N2—C10—C9	111.8 (2)	H24A—C24—H24B	109.5
N2—C10—C11	120.3 (2)	C23—C24—H24C	109.5
C9—C10—C11	128.0 (2)	H24A—C24—H24C	109.5
C10—C11—H11A	109.5	H24B—C24—H24C	109.5
C10—C11—H11B	109.5	N3—C25—H25A	109.5
H11A—C11—H11B	109.5	N3—C25—H25B	109.5
C10—C11—H11C	109.5	H25A—C25—H25B	109.5
H11A—C11—H11C	109.5	N3—C25—H25C	109.5
H11B—C11—H11C	109.5	H25A—C25—H25C	109.5
N1—C12—H12A	109.5	H25B—C25—H25C	109.5
N1—C12—H12B	109.5	O4—C26—C22	124.5 (3)
H12A—C12—H12B	109.5	O4—C26—H26	117.8
N1—C12—H12C	109.5	C22—C26—H26	117.8
			
C8—N1—N2—C10	0.3 (3)	C8—C9—C13—O2	173.5 (2)
C12—N1—N2—C10	−179.72 (19)	C10—C9—C13—O2	−9.2 (4)
C21—N3—N4—C23	−0.5 (3)	C21—O3—C14—C19	−13.2 (3)
C25—N3—N4—C23	−179.5 (2)	C21—O3—C14—C15	167.3 (2)
C8—O1—C1—C6	−14.8 (3)	C19—C14—C15—C16	−1.9 (4)
C8—O1—C1—C2	167.7 (2)	O3—C14—C15—C16	177.68 (19)
C6—C1—C2—C3	0.6 (4)	C14—C15—C16—C17	1.1 (4)
O1—C1—C2—C3	178.1 (2)	C15—C16—C17—C18	−0.1 (4)
C1—C2—C3—C4	0.0 (4)	C16—C17—C18—C19	−0.1 (4)
C2—C3—C4—C5	−0.4 (4)	C16—C17—C18—C20	179.7 (2)
C3—C4—C5—C6	0.3 (4)	C15—C14—C19—C18	1.6 (4)
C3—C4—C5—C7	−179.1 (2)	O3—C14—C19—C18	−177.9 (2)
C2—C1—C6—C5	−0.7 (4)	C17—C18—C19—C14	−0.6 (4)
O1—C1—C6—C5	−178.0 (2)	C20—C18—C19—C14	179.6 (2)
C4—C5—C6—C1	0.2 (4)	N4—N3—C21—O3	−176.2 (2)
C7—C5—C6—C1	179.6 (2)	C25—N3—C21—O3	2.7 (4)
N2—N1—C8—O1	176.58 (19)	N4—N3—C21—C22	−0.4 (3)
C12—N1—C8—O1	−3.4 (4)	C25—N3—C21—C22	178.5 (2)
N2—N1—C8—C9	−0.9 (3)	C14—O3—C21—N3	−91.8 (3)
C12—N1—C8—C9	179.1 (2)	C14—O3—C21—C22	93.5 (3)
C1—O1—C8—N1	103.3 (3)	N3—C21—C22—C23	1.0 (3)
C1—O1—C8—C9	−79.9 (3)	O3—C21—C22—C23	176.2 (2)
N1—C8—C9—C10	1.1 (3)	N3—C21—C22—C26	−178.3 (2)
O1—C8—C9—C10	−176.1 (2)	O3—C21—C22—C26	−3.2 (5)
N1—C8—C9—C13	179.0 (2)	N3—N4—C23—C22	1.2 (3)
O1—C8—C9—C13	1.8 (4)	N3—N4—C23—C24	178.74 (19)
N1—N2—C10—C9	0.4 (3)	C21—C22—C23—N4	−1.4 (3)
N1—N2—C10—C11	−179.8 (2)	C26—C22—C23—N4	177.9 (2)
C8—C9—C10—N2	−0.9 (3)	C21—C22—C23—C24	−178.7 (2)
C13—C9—C10—N2	−178.7 (2)	C26—C22—C23—C24	0.6 (4)
C8—C9—C10—C11	179.3 (2)	C21—C22—C26—O4	−177.9 (3)
C13—C9—C10—C11	1.6 (4)	C23—C22—C26—O4	2.9 (4)

### Hydrogen-bond geometry (Å, °)

**Table:**

*D*—H···*A*	*D*—H	H···*A*	*D*···*A*	*D*—H···*A*
C12—H12A···O4^i^	0.98	2.57	3.488 (3)	157
C15—H15···O2^ii^	0.95	2.58	3.315 (3)	134

Symmetry codes: (i) −*x*+1, −*y*, −*z*; (ii) *x*−1, *y*−1, *z*.
